# “If you work alone on this project, you can’t reach your target”: unpacking the leader’s role in well-performing teams in a maternal and neonatal quality improvement programme in South Africa, before and during COVID-19

**DOI:** 10.1186/s12913-023-10378-x

**Published:** 2023-12-08

**Authors:** Willem Odendaal, Terusha Chetty, Mark Tomlinson, Ameena Goga, Yages Singh, Shuaib Kauchali, Carol Marshall, Xanthe Hunt

**Affiliations:** 1 HIV and Other Infectious Diseases Research Unit, South African Medical Research Council, Francie Van Zijl Drive, Parow Valley, Cape Town, Western Cape South Africa; 2 Department of Psychiatry, Stellenbosch University, Francie Van Zijl Drive, Tygerberg, Cape Town, Western Cape South Africa; 3 Discipline of Public Health Medicine, School of Nursing and Public Health, University of KwaZulu-Natal, Durban, KwaZulu-Natal South Africa; 4 Institute for Life Course Health Research, Stellenbosch University, Franzi Van Zijl Drive, Tygerberg, Cape Town, Western Cape South Africa; 5 School of Nursing and Midwifery, Queens University, Belfast, UK; 6 Department of Paediatrics and Child Health, University of Pretoria, Steve Biko Academic Hospital, Pretoria, Gauteng South Africa; 7 Department of Paediatrics and Child Health, Nelson Mandela School of Medicine, University of KwaZulu-Natal, Durban, South Africa; 8 South African National Department of Health, Voortrekker Road, Pretoria, Gauteng South Africa

**Keywords:** Contexts and implementation processes, Low-and-middle income country, Maternal and neonatal health, Qualitative evaluation, Quality improvement, Team leader attributes, Team performance

## Abstract

The South African National Department of Health developed a quality improvement (QI) programme to reduce maternal and neonatal mortality and still births. The programme was implemented between 2018 and 2022 in 21 purposively selected public health facilities. We conducted a process evaluation to describe the characteristics and skills of the QI team leaders of well-performing teams. The evaluation was conducted in 15 of the 21 facilities. Facilities were purposively selected and comprised semi-structured interviews with leaders at three time points; reviewing of QI documentation; and 37 intermittently conducted semi-structured interviews with the QI advisors, being QI technical experts who supported the teams. These interviews focused on participants’ experiences and perceptions of how the teams performed, and performance barriers and enablers. Thematic data analysis was conducted using Atlas.ti. Variation in team performance was associated with leaders’ attributes and skills. However, the COVID-19 pandemic also affected team functioning. Well-performing teams had leaders who effectively navigated COVID-19 and other challenges, who embraced QI and had sound QI skills. These leaders cultivated trust by taking responsibility for failures, correcting members’ mistakes in encouraging ways, and setting high standards of care. Moreover, they promoted programme ownership among members by delegating tasks. Given the critical role leaders play in team performance and thus in the outcomes of QI programmes, efforts should focus on leader selection, training, and support.

**Supplementary Information:**

The online version contains supplementary material available at 10.1186/s12913-023-10378-x.

## Background

Quality improvement (QI) in healthcare is the process by which healthcare workers (HCWs) reorganise care and test changes on a small scale, using existing resources to improve care and health outcomes [1, 2]. QI programmes are well established in high, low—and middle-income countries (LMICs) [3, 4]. There are several QI models, including *Lean*, *Sigma,* and *Six Sigma* [5, 6]. The *Plan-Do-Study-Act* (PDSA) cycle is an often-cited model [7], applied in maternal and neonatal healthcare (MNH) [8, 9]. It comprises rapid, iterative cycles of: *Plan* concerns developing a Quality Improvement Plan (QIP) to fix the identified problem; *Do* is implementing the QIP; *Study* is assessing the QIP outcomes; and *Act* is when the team decides to either adopt, i adapt, or abandon the QIP [10].

There is consensus in several systematic reviews that the mixed effectiveness results of QI programmes are driven by implementation contexts and processes [11, 12]. It includes resource availability, data infrastructure, facility size, readiness for change, and methodological adaptations [3, 13, 14]. It also refers to interpersonal relationships between the actors in the health system within which the programme is implemented [15], that affect QI outcomes [13]. Key amongst these relationships is those between the QI team leader (henceforth interchangeably referred to as ‘leader’) and team members [15, 16].

HCWs generally deliver care in teams and therefore QI effectiveness is related to team effectiveness, with the team leader instrumental in how well the team performs [17, 18]. Though the late 1980’s saw a focus on leadership in public health [19], many of its current theories share constructs developed in other fields, for e.g. a leader’s integrity and self-confidence are as desirable in healthcare [20], as in commerce [21]. General leader characteristics and skills that apply to the QI leader include creating a shared vision in the team [22]. This ensures that members own the programme [23], which increases its chance of success [23, 24]. This ownership is further fostered when the leader is democratic and consultative [25, 26], motivating members to equally contribute towards shared goals [27, 28]. When it is only leaders who receive training, as often reported in QI programmes [29, 30], their ability to transfer these skills is key in how well-versed teams become in using the intervention tools [31]. In all this, the leader’s communication skills are central [28].

There are two characteristics and skills specific to a QI leader. Firstly, they must prioritise team members’ learning by encouraging them to identify and correct service errors [17], and cultivate honest self-reflection on the standard of their work [31, 32]. In assessing the learning behaviour of hospital-based QI teams in Ghana, it was found that team learning happens best with a leader who is not punitive [17], but creates emotional safety by valuing members’ input and celebrating their achievements [9]. A culture of continued learning amongst team members is important to improve healthcare and the leader central in creating this culture [33]. Team members respond positively to a leader’s feedback when the leader is someone that they trust [24, 34]. Secondly, the leader must possess the necessary technical QI skills [35]. The stages of a PDSA cycle have much in common with the stages of a scientific experimental study [5]. This demands a leader with some understanding of the rigor that QI requires, and the technical abilities to interrogate data, do a root cause analysis, and maintain run charts [2, 13, 35].

Despite progress to reduce maternal and neonatal mortality and still births in South Africa (SA), [36–38], the country is not on target to meet the Sustainable Development Goals (SDGs), for instance, its institutional maternal mortality ratio (iMMR) of 134 deaths per 100 000 live births in 2017 [39], is notably higher than the SDG maternal mortality target of 70/100,000 [40]. The COVID-19 pandemic exacerbated this situation with an estimated 22.7% increase in iMMR and a 4.8% increase in institutional neonatal mortality, between March and December 2019 compared to the same period in 2020 [41]. There is consensus that many of these deaths could be avoided if healthcare was improved [40, 42]. It is estimated that strengthening midwives’ capacity in low—and middle—income countries may prevent 41%, 39% and 26% of all maternal deaths, neonatal deaths, and stillbirths, respectively [43].

To that end, the SA National Department of Health (NDoH) launched the *Mphatlalatsane* (meaning ‘the bright star before dawn’) *Initiative* between 2018 and 2022. *Mphatlalatsane* was a multi-partnered programme to reduce maternal and neonatal mortality, and still births by up to 50% in 21 purposively selected facilities in SA [30]. We conducted a longitudinal, qualitative process evaluation of the *Mphatlalatsane* QI teams with three data collection time points over the 39 months that the initiative was implemented. The aim of the evaluation was to assess the implementation processes and contextual factors that shaped the performance of the QI teams in the participating facilities. In this paper we explore the key leader attributes associated with how well a team performed.

## Methods

Our evaluation is part of a larger mixed-methods evaluation [44] of *Mphatlalatsane*. Ethical approval was obtained from respectively the South African Medical Research Council and Stellenbosch University in South Africa.

### Setting

**Fig. 1:**
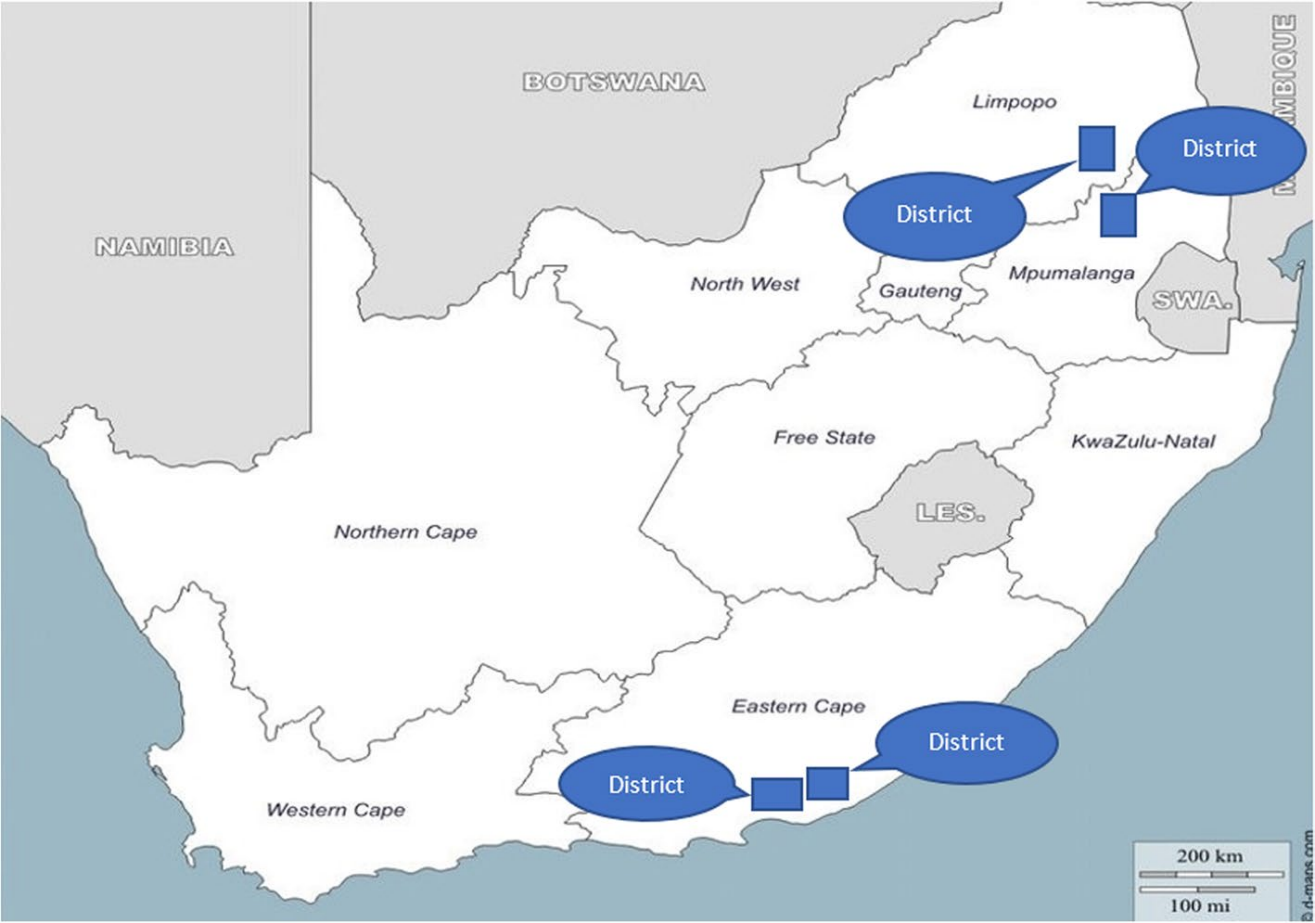
Mphatlalatsane implementation districts

The *Mphatlalatsane* facilities are situated in four health districts across three provinces in SA, Mpumalanga, Limpopo, and the Eastern Cape. (Fig. 1; see Additional file 1 for the districts’ socio-economic and health indicators).

The NDoH purposively selected seven facilities per province to reflect the referral pathways within the districts. These facilities comprised of two primary healthcare (PHC) clinics, two community health centres (CHCs), two district hospitals, and one regional hospital. The facilities represented average to below-average performance on selected perinatal indicators of iMMR. One district is mainly urban [45], and the others mostly rural [45–47]. *Mphatlalatsane* was implemented through the respective provincial departments of health and district offices. Each facility’s management nominated two or three senior HCWs to be trained by the Institute for Healthcare Improvement on the PDSA model [30, 48]. Districts 1 and 2 had training in September 2019 with District 1 teams receiving two more trainings before the COVID-19 lock-down in March 2020. Districts 3 and 4 facilities attended one training in February 2020. Upon returning from training, the trainees and facility management selected the team leader from those who attended the training and recruited team members from the facility staff. There were no uniform selection criteria used in selecting the HCWs for training or selecting the leader. The size of the teams varied from four to 12 members. The Clinton Health Access Initiative, the *Mphatlalatsane* partner coordinating its implementation, appointed QI advisors (hereafter also referred to as ‘advisors’), who provided technical QI support and mentoring to the teams. There was one advisor for District 1 and 2 respectively, and one for Districts 3 and 4 jointly.

### Sampling

We purposively selected 15 of the 21 facilities (Table 3 below) of which 14 participated in the evaluation. The one that did not participate felt their QI activities were too disrupted during COVID-19 for meaningful participation. These facilities included rural and urban settings and represented the range of *Mphatlalatsane* facility types. We also used a pre-*Mphatlalatsane* readiness assessment conducted by the NDoH and selected better-prepared and less-prepared facilities. We consulted with the advisors to ensure that the selected facilities were a true representation of all *Mphatlalatsane* facilities. With one facility having two teams, there were 15 teams from the 14 facilities participating in the evaluation.

### Participants

At the start of *Mphatlalatsane,* there were three advisors appointed. Over the implementation period, all three of them resigned. With only two of them replaced, there were five QI advisors during the implementation period, and all participated in the evaluation. We recruited all team leaders (hence on, also referred to as ‘leaders’), across the 15 teams. Given leader change in two teams, 17 leaders participated.

### Data collection

The evaluation, conducted between February 2020 and November 2022, comprised the following.

#### QI advisor interviews and their documentation

We conducted 37 advisor interviews. The lead author briefed them about the evaluation, and obtained their signed, informed consent before the first interview. The interviews were mostly joint interviews with more than one advisor present, but at times with individual advisors when all were not available. All interviews were conducted on Microsoft Teams (https://www.microsoft.com/en/microsoft-teams/group-chat-software). The advisors shared their perceptions and experiences on team functioning, implementation progress, and implementation enablers and barriers over time. Though we explored these issues in all interviews following Interview 1 (Additional file 2a), we tailored Interviews 2 to 37 to include follow-up questions from previous interviews. They also made their programme documentation available to the research team.

#### Team leader interviews and their documentation

We interviewed 16 of the 17 leaders (Table 1) at three time points: May 2021 (Timepoint 1), September 2021 (Timepoint 2), and September 2022 (Endpoint). We opted for multiple data collection timepoints to explore implementation progress and changes over time, for example if there were any changes to how the PDSA model was implemented. Since we also recruited team members as evaluation participants, we requested individual interviews with the leaders and members. We had 35 leader interviews, of which 24 were with leaders individually; by leaders’ choice, team members joined in the other 11 leader interviews. The interviews were on average 42 min long.

**Table 1:** Team leader interviews

	**District 1** (6 facilities)	**District 2** (5 facilities)	**District 3** (1 facility)	**District 4** (2 facilities)	**Total**
Leader participants	7	6	2	2	**17**
Leader individual interviews	10	9	2	3	**24**
Leader interviews with members present	3	5	2	1	**11**

We conducted a site visit prior to Timepoint 1 to recruit the leaders. After briefing them about the evaluation, we obtained their signed informed consent. The interviews were conducted in-person at the facilities, in an office the leaders made available, and at a time convenient to them. The interviews focused on their views and experiences of implementation enablers and barriers, their role, and teamwork. Though we explored these issues in all interviews following the Timepoint 1 (Additional file 2b), we tailored the Timepoint 2 and Endline interviews to include specific follow-up questions from previous interview. Leaders also made their QI documentation available.

All interviews were conducted in English, audio recorded, and transcribed. The lead author (WO) collected all the data.

#### Fieldwork journal

WO kept a fieldwork journal in which he recorded his fieldwork reflections.

We also recruited and interviewed 47 HCWs who served as QI team members. The data collection and analysis were the same as described in this paper. The members’ demographic information and perceptions and experiences of the leader’s role in team performance will be reported in a forthcoming publication.

### Analysis

The lead author verified the accuracy of all transcripts against the recordings. The transcripts, program documentation and fieldwork journal were analysed using Atlast.ti, 8.1 (https://atlasti.com/). We applied the thematic analysis method developed by Graneheim and Lundman [49]. The lead author coded five Timepoint 1 transcripts and the first three advisor interviews and discussed it with two senior members (XH, TC) of the research team. The code list was amended, and the remaining data coded. We amended the list with new codes emerging from Timepoint 2 and Endpoint data. The codes were grouped into sub-themes and these into three overarching themes. During regular team meetings the lead author and the mentioned two senior research team members refined the coding and analysis. We did not analyse the data from members who joined the leader interviews.

We defined ‘team performance’ as how well a team made use of the QI methodology over the implementation period, that started with the 1^st^ training a team received, until November 2022. For Districts 1 and 2, the implementation period began in September 2019, and for Districts 3 and 4, it commenced February 2020. We used four criteria to determine team performance:

- *Leadership*: evidence of a team structure with a leader;
- *Sustained activities*: sustained QI activities during the COVID-19 period between August 2020 and March 2021 (QI activities were suspended between March to July 2020);
- *Attitude*: the leader’s attitude towards QI and perceptions regarding the team’s performance; and
- *QI maturity*: the extent to which the team was perceived by the advisor and WO to function without constant advisor support towards the final months of the implementation period.

‘Well-performing teams’ were those for whom there was evidence of a functional team with a leader; sustained QI activities between August 2020 to March 2021; had leaders who were QI enthusiasts and positive about their teams’ performance; and ‘QI matured’. “Less well performing teams” were teams who did not meet one or more of the four criteria. The lead author confirmed his assessments of QI maturity for Districts 1, 3 and 4 teams with the respective advisors in September 2022. Maturity for the District 2 teams was not confirmed with the advisor as she had left the programme before the confirmation could happen.

## Results

We present the results across four themes: *Theme One* details the importance of the leader; *Theme Two,* the challenges they faced; *Theme Three* presents QI specific leader characteristics and skills; and *Theme Four* covers how successful leaders promoted members owning the programme.

### Participant characteristics

#### Team leaders

All were female midwives, except for one who was a doctor. In two CHCs and in the clinics, they were facility operational managers (OPMs). The leaders in the other CHCs and all the hospitals were ward OPMs in the maternity unit. They were experienced HCWs with on average 28 years in nursing (Table 2). In Districts 1—3 they had on average eight years’ management experience and in District 4, two years. The average number of years at their facilities was 20 years, ranging between one and 33 years. The two leader replacements, one retired and one transferred to a non-participating facility, were in Districts 1 and 2 respectively.

**Table 2:** Team leaders’ healthcare and management experience

	**Average years in nursing** (range)	**Average years as manager** (range)	**Average years at facility** (range)
**District 1** (8 leaders)	31 (8–42)	8 (1–22)	20 (1–33)
**District 2** (6 leaders)	31 (21–37)	11 (2–24)	20 (10–30)
**District 3** (1 leader)	38	11	32
**District 4** (2 leaders)	16 (9–22)	2 (2)	10 (2–18)
**Average** (17 leaders)	28 (8–42)	8 (0–21)	20 (1–33)

In comparing these characteristics between leaders of well-performing and less well performing teams, it was found that the former had slightly more nursing experience (29 years) than leaders of less well performing teams (25 years). The same trend was observed regarding their management experience, 10 years compared to 7 years, respectively for leaders of well-performing and less well performing teams, and for the number of years at the facility: leaders of well-performing teams were on average 20 years at their respective facilities, compared to the 16 years for leaders of less well-performing teams.

#### QI advisors

All three initially appointed advisors resigned over the implementation period: the advisor who managed Districts 3 and 4 resigned in March 2021; the District 1 advisor in July 2021, and the District 2 advisor, August 2022. The District 3 and 4 teams had no advisor for four months, as the replacement advisor started working in August 2021. The District 1 teams were without an advisor for one month, as the new advisor was appointed in September 2021. The District 2 teams remained without an advisor from August to December 2022. Four advisors had between 17 months and 15 years’ experience of QI mentoring and trained by well-known QI institutions.

#### QI teams

We assessed six of the 15 teams as well-performing (Table 3): four in District 1, two in District 2, and none in Districts 3 and 4. The detail of our assessment can be found in Additional file 3.

**Table 3:** Participating facilities and well-performing teams by district and by facility type

Type of facility	District 1	District 2	District 3	District 4	Total
Regional hospital (well-performing teams)	1 (1)	1* (0)	d (0)	§ -	**2** **(1)**
District hospital (well-performing teams)	2 (0)	1 (0)	§ -	1 -	**4** **(0)**
CHC (well-performing teams)	2 (2)	2 (1)	1 (0)	§ -	**5** **(3)**
PHC clinic (well-performing teams)	1 (1)	1 (1)	§ -	1 (0)	**3** **(2)**
**Total (well-performing teams)**	**6** **(4)**	**5** **(2)**	**1** **(0)**	**2** **(0)**	**14** **(6)**

*CHC* Community healthcare centre, *PHC clinic* Primary healthcare clinic

^*^Facility with two teams

^d^Declined participation

^§^No facility selected

### Theme One: The importance of the team leader

#### Importance of the team leader

Most leaders, and all advisors, agreed that the leader is central to team performance. As one leader suggested, the leader determines “above 70%” of team performance” (Leader, Well-performing team 1). When probed on why the leader is important, advisors said that it had to do with there being someone to take charge, provide guidance, and create energy for team activities:

“This is the person who should be able to say: “Guys, now we are meeting for our QI meeting”; “Guys, we have to brainstorm.” … if the team leader is not doing that, nobody will do it in the facility.” (Advisor 4)

The importance of leadership was also borne out in two less well-performing facilities. In one, the initially appointed—and replacement leaders respectively, moved to other facilities. With no one stepping up as leader, the team disintegrated. In the second case, the originally selected leader unexpectedly left *Mphatlalatsane*, resulting in a dead stop of QI activities until the replacement leader was appointed:

“Then it [*Mphatlalatsane*] was abandoned on the way because our team leader just went out of the project … we didn’t have a leader and we didn’t meet.” (Replacement leader, Less well-performing team 5)

### Theme Two: Team leader challenges

There were two challenges all leaders faced. Firstly, they suffered staff shortages and described how it resulted in work overload, which made it difficult for teams to attend to QI activities. As one leader noted:

“We need more staff, especially because now we depend more on the Comserves [HCWs doing their community service], and the permanent staff are less.” (Leader, Less well-performing team 2)

The second challenge was ensuring staff buy-in. Leaders had to deal with members’ skepticism and resistance about doing something new, but also because members were already overstretched because of staff shortages:

“They thought that we were abusing the power, but they didn’t see the way we were seeing it.” (Leader, Less well-performing team 6)

“You see, when you are about to bring change, there is this resistance, a shortage of staff and more work to them.” (Leader, Well-performing team 6)

The third challenge was the COVID-19 pandemic, with its wide-ranging impact on the leader and team morale. It caused emotional trauma for all HCWs, whether they worked in a big regional hospital or small, rural PHCs. This was attested to by many leaders who had similar experiences as this leader:

“The manner in which Covid came, we were so afraid. It was difficult for us to come on duty every day because you never knew when you came in if you will be able to go out alive.” (Leader, Well-performing team 2)

During the first five months of COVID-19, the teams abandoned their QI activities because they were tasked with curbing the pandemic. The advisors observed that the facilities where COVID-19 patients were treated, suffered more severe service disruptions than referring facilities. All six hospitals and the District 3 CHC served as treatment centres, and the disruptions the leaders there experienced, were probably a bigger challenge than what the referring CHCs and PHC clinics experienced.

“ … because remember, they [treatment centres] now had to set up isolation sites. They had to move staff around to those isolation [sites] … hence now they’re only focused on Covid and other things they put on hold … ” (Advisor 2)

A final challenge the CHCs and hospitals experienced because they rendered 24-h shifts, was ensuring that the night shift members were informed about, and adhered to, the QIP protocols developed by the day shift members. It was also challenging to give night shift members feedback on how well they were implementing the QIP.

“We don’t have the time to meet. Because you’ll find that four professional nurses are at night, and you’ll find that most of them are in the team. Then we don’t have time [to meet] and we are working different shifts.” (Leader, Less well-performing team 5)

### Theme Three: Quality improvement specific leader characteristics and skills

#### Quality improvement enthusiasts

The QI methodology resonated with all the leaders whose teams performed well, and these leaders said: “I am so much in love with it”; “*Mphatlalatsane* was an eye-opener because previously there were things that we were not doing correctly.”; and “Its enriching my mind. Its enriching my staff.”

There were some leaders who returned from the QI training as ‘QI converts’, but for the majority it was the drastic improvements from their first QIP that got them excited about QI, represented in what this leader had to say:

“The thing is, before, we were not aware that there were methodologies and whatever that we can use to improve our performance and to improve the quality in our facility. But since *Mphatlalatsane* came in … we have seen quite a massive improvement.” (Leader, Well-performing team 3)

This commitment resulted in a positive attitude to solve some of the challenges all leaders faced. In contrast to the leader who could not manage night shift staff in the team, a leader from a well performing team resolved the challenge:

“In the morning I will check them [the files the member working night shifts completed] and keep them. When she comes [back on day shift], I say, sister X, you didn’t correct here, why?” (Leader, Well-performing team 2)

These attituded were in stark contrast with how some leaders from less well-performing teams, viewed QI:

“… [we are not] statisticians, for the lack of a better word … isn’t there somebody else that’s supposed to do that? We’re clinicians.” (Leader, Less well-performing team 7)

It was also not clear why for some leaders “QI is not their thing.” (Advisor 4), however their attitude towards QI set the tone for less well-team performance for the duration of *Mphatlalatsane* implementation:

“My challenge now is that I don’t have time to monitor each and everything … I just monitor it [QI] once in a month when I’m doing the statistics only. But the [QI] project itself, I don’t have that time to attend to it.” (Leader, Less well-performing team 5)

Solutions involving more staff and with financial implications, such as installing an extra phone in the labour ward, had to have the blessing of the senior facility management. It is not surprising that the QI enthusiastic leaders more frequently reported getting that support, than leaders from less-well performing teams.

#### View QI as routine care

QI enthusiastic leaders did not experience QI is additional to routine healthcare (first quote) but viewed it as a tool to do what they were supposed to do, better. The contrasting view from a leader of a less-well performing team (second quote), illustrates that for her it was just a burden:

“… but this [QIP] was something that has been communicated all along … that every childbearing woman, they have to test for pregnancy even if she’s coming to the outpatients’ department ...” (Leader, Well-performing team 2)

“… if I ignore it [*Mphatlalatsane*], it will go away and I won’t have to look at it, that was the approach we had.” (Leader, Less-well performing team 7)

It is therefore not surprising that most leaders from well-performing teams reported embedding their QIPs into routine practices in their facilities:

“I can’t say [*Mphatlalatsane*] is work because it’s part of our daily practice.” (Leader, Well-performing team 5)

#### QI technical capabilities

Leaders’ QI enthusiasm was coupled with having the necessary technical skills to interrogate routine data to identify service delivery problems; do a root cause analysis of service delivery problems; and implement PDSA cycles:

“Because what’s been great to hear from Facility Y, is a clinician [team leader] saying: “I’ve done a fishbone, I’ve done root cause analysis.” So, she didn’t just tell them [members] about the project … but she’s also teaching them about the actual QI methodology.” (Advisor 1)

Though some leaders of less well-performing teams had a sound understanding of the methodology and the technical skills to conduct a QIP, there were several who struggled with the concept:

“I’m running in circles and not winning with any of the change ideas [QIPs]” (Leader, Less well-performing team)

#### Foster a learning culture 

Leaders of well-performing teams displayed several characteristics that allowed members to trust them and in turn cultivated a learning culture in their teams. Firstly, they assumed responsibility for the healthcare under their command, acknowledged their own failures, and were open to critique:

“Like we said, it’s [the leader] somebody who should be proactive, passionate, open to criticism and accepting those criticisms as constructive. Ja, we are subject to being criticised.” (Leader, Well-performing team 1)

Secondly, they accepted that members could, and did, make mistakes with QIP implementation, but this they addressed it in a positive manner.

“So, we call them, all the midwives, then we say: “Okay, guys, here are files here, check all these files, almost 70 percent is [incomplete] and it can’t be.” … Some, they were saying: “Ja, we are afraid of sister so-and-so …”. So, we’re getting each idea, what was their problem.” (Leader, Well-performing team 2)

Yet, they also acknowledged and celebrated team achievements, and when this happened: “… then they [members] become so excited.” (Leader, Well-performing team 3).

In addition, they acknowledged that MNH services at times made complete implementation of the QIP hard or impossible, because “maternal and neonatal, [is] a fast-paced environment” (Advisor 3), where managing a life-threatening situation for mother or baby took precedence over the QIP:

“Sometimes it’s in the morning when we arrive, we’ll find that there are three or four in labour, some want to deliver now, so it becomes so difficult to say let’s come and sit … but afterwards when it’s quiet, we take those files and check.” (Leader, Well-performing team 2)

Thirdly, though they refrained from being punitive towards member errors, they were assertive when needed.

“After identifying problems, especially if the problems are so common, we have to meet and say: “Guys, what do you think is the problem? Why are these files like this?” Then they will say: “No, maybe it was too busy in the outpatients’ department, and we didn’t manage.” I said: “No, you have to do your thing at the right time.”” (Leader, Well-performing team 2)

Fourthly, they were also very persistent, which an advisor described as leaders’ “tenacity”, that members kept to the agreed QIP protocols. There was nothing magical in how this was done, other than daily monitoring of implementation, and as important, providing regular feedback to the team:

“… it’s good for the leader to go and assess and see the source document. You cannot hear from them – you [have to] see the source document. You check that source document and if you see that they are not doing well on this one, you talk to them.” (Leader, Well-performing team 5)

### Theme Four: Leader skills to promote member ownership

Successful leaders displayed more general leadership skills to facilitate members’ ownership of the programme, than leaders from less well-performing teams.

The first skill related to overcoming members’ initial reluctance towards QI. Some leaders of well-performing teams secured member buy-in by: “… not forcing them but by giving them what they could see that is working, then they started to continue.” (Leader, Well-performing team 6). Another leader overcame resistance by ensuring members had a shared vision of what they wanted to achieve:

“… if they do not know how and why, then they resist because they don’t see that vision.” (Leader, Well-performing team 6)

In contrast, less well-performing team leaders who had misgivings and negative experiences about securing team buy-in, linked it to their own lack of confidence in understanding the QI methodology.

“… in terms of implementing it [the training] back at your facility, it is sometimes difficult as someone who has just learnt something new, to now sort of inspire every person at the facility to learn everything.” (Leader, Less well-performing team 1)

Successful leaders further promoted team ownership by delegating duties in the team and valuing members’ input. One leader pointed out that it is important to value junior members’ input as much as the seniors’:

“Even the junior nurses at times tell us that “No, that one is not going well.”, and we should be doing 1, 2, 3. We don’t look down on them … We say: “Okay, there’s a complaint, the juniors are saying 1, 2, 3 is not done very well.”, then we discuss how best we can change it.” (Leader, Well-performing team 1)

The importance of delegation is illustrated in the change in an advisor’s assessment of one of her teams. At first the team was not performing well which she attributed to the leader doing the work on her own. Then in a later interview she observed an improvement in team performance, and when asked what brought it about, she concluded as follows:

“… what I noticed, especially during Covid, their OPM [leader] was now delegating duties to other team members … Ja, it must be that because that’s the only thing that has changed.” (Advisor 2)

In addition to securing buy-in, delegating tasks, and valuing members’ input, leaders of well-performing teams were attuned to personality differences in their teams:

“You can be democratic, or you can be autocratic, it will be according to the people that you are working with.” (Leader, Well-performing team 5)

## Discussion

This study provides a deep understanding of QI team leaders’ and advisors’ views and experiences regarding the leader’s role in the implementation of a MNH QI programme in resource-constrained settings. It details how the leaders set performance standards and guidance to their teams, and the challenges leaders faced, notably COVID-19 disruptions. The evaluation highlights key leader traits and skills associated with well-performing teams. These include, being passionate about QI; having the necessary technical skills; and establishing a trusting relationship within the team. A trusting climate resulted from leaders who could admit their own failures; correct members’ errors in a constructive manner yet be assertive and persistent in what they expected from the team. Ensuring that members take ownership of their QI activities required leaders who could delegate tasks and be sensitive to personality differences amongst team members.

### Team leader characteristics and skills

Our participants’ view that leadership is central to a QI team’s performance, confirms the result from a study measuring the effectiveness of QI teams who addressed mental health in the Netherlands [50]. The authors found that across the 29 teams being assessed, that around 50% of successful outcomes was associated with how inspirational the leader was [50]. Our results highlighted several characteristics and skills that are specific to QI programmes. It confirmed that a leader who is enthusiastic about the methodology [51]; has the technical skills to run QIPs [35]; views QI as a tool to support what HCWs are supposed to do [52]; and can foster a learning culture amongst team members [13, 53], equate to a well-performing team. We also concur that more general leader traits can be associated with well-performing teams, including getting member buy-in [51]; being able to delegate tasks and valuing member contributions [23, 24]; and an awareness that members had different personalities and needed to be treated differently in certain circumstances. [51] Yet, the leader’s enthusiasm must be supported by senior personnel in the facility, as cited in a QI programme that improved TB-HIV services, as one of the reasons for its success [19].

Our finding that the leaders of well-performing teams displayed tenacity in keeping the teams to task, despite competing priorities [51], is in line with a study which found a significant and positive relationship between the length of QI involvement and its success [11]. From being QI naïve to becoming a proficient QI implementer, takes time [54]. We did not find literature on how to identify an enthusiastic QI leader, but QI is a data-driven methodology [1, 5]. QI programme managers will do well to ascertain potential leaders’ aptitude to interrogate and use data before appointing them.

This study confirmed the importance of leaders’ need to balance their acceptance that to err is human [13], and therefore that members will make mistakes, with assertiveness and persistence once the team agreed on how a QIP should be implemented [51]. In doing this, the softer interpersonal skills such as openness to critique [53], and not having a ‘blame and shame’ attitude towards members who make mistakes [17], become important tools for a leader to optimise team performance.

Though we found that the leaders of well-performing teams had more nursing and management experience, and had been working longer at their respective facilities, our data did not allow quantifying the effective it may have had on their leadership.

### Contexts and implementation processes that shaped team performance

In an earlier manuscript (under review), we described implementation processes and contexts, not related to the leader, which shaped the intervention uptake amongst the *Mphatlalatsane* QI programmes. This includes an example of a less well-performing team that was part of a larger facility context that was completely dysfunctional because of staff strikes and poor working conditions. None of the leaders of well-performing teams in our evaluation suffered this, and had it been the case for them, it may have turned out to have the same effect on their team’s performance. An implementation barrier we detailed in the manuscript under review, is that Districts 3 and 4 teams had only one month of implementation and advisor support after training when COVID-19 became an issue. This must have contributed as much as the leader, if not more, to why these teams did not perform as well as their Districts 1 and 2 colleagues. With this we acknowledge that leaders of less well-performing teams may have been exposed to contexts and processes they could do little about, but that we were unaware of. In these cases, less-well team performance may have been more a function of contexts and processes, than of the leader.

Our data does not allow us to weigh the contribution of each leader attribute in team performance, but we are able to suggest that being a committed QI enthusiast [51], is the main trait a leader must have if the team is to be successful. This enthusiasm turned into a positive, self-enforcing cycle: the more enthusiastic the leader, the more effort they put into a QIP; the bigger the effort, the more likely the QIP would show improvement in service delivery; and the bigger the improvement, the more the leader’s enthusiasm became. It has been reported, as was found in *Mphatlalatsane,* that some QIPs can result in large improvements [55]. Those managing QI programmes may ignite QI enthusiasm amongst leaders by guiding them to start off with QIPs that are likely to yield a large improvement.

Our results have several implications for policymakers of which we consider the following one as the most fundamental to a successful QI programme. If senior facility management, and subsequently, the HCWs who will be recruited as team leaders, are to accept QI as a tool to improve the quality of standard care, and not as additional to standard care [56], then QI should be endorsed as policy [11]. Such endorsement may result in cascading buy-in downwards across the different management levels, ending with QI leaders who know they are supported across all levels. When frontline HCWs experience such high-level support, it motivates them to use their experience and knowledge to provide quality healthcare [57].

For those healthcare managers who will be charged with setting up and managing a QI programme, we offer the following recommendations (Table 4), pertaining to identifying, training, and mentoring a MNH healthcare worker into a competent QI team leader. These recommendations are based on our sensemaking of the views and experiences the Mphatlalatsane team leaders and QI advisors on what is required to become a proficient QI team leader.

**Table 4:**
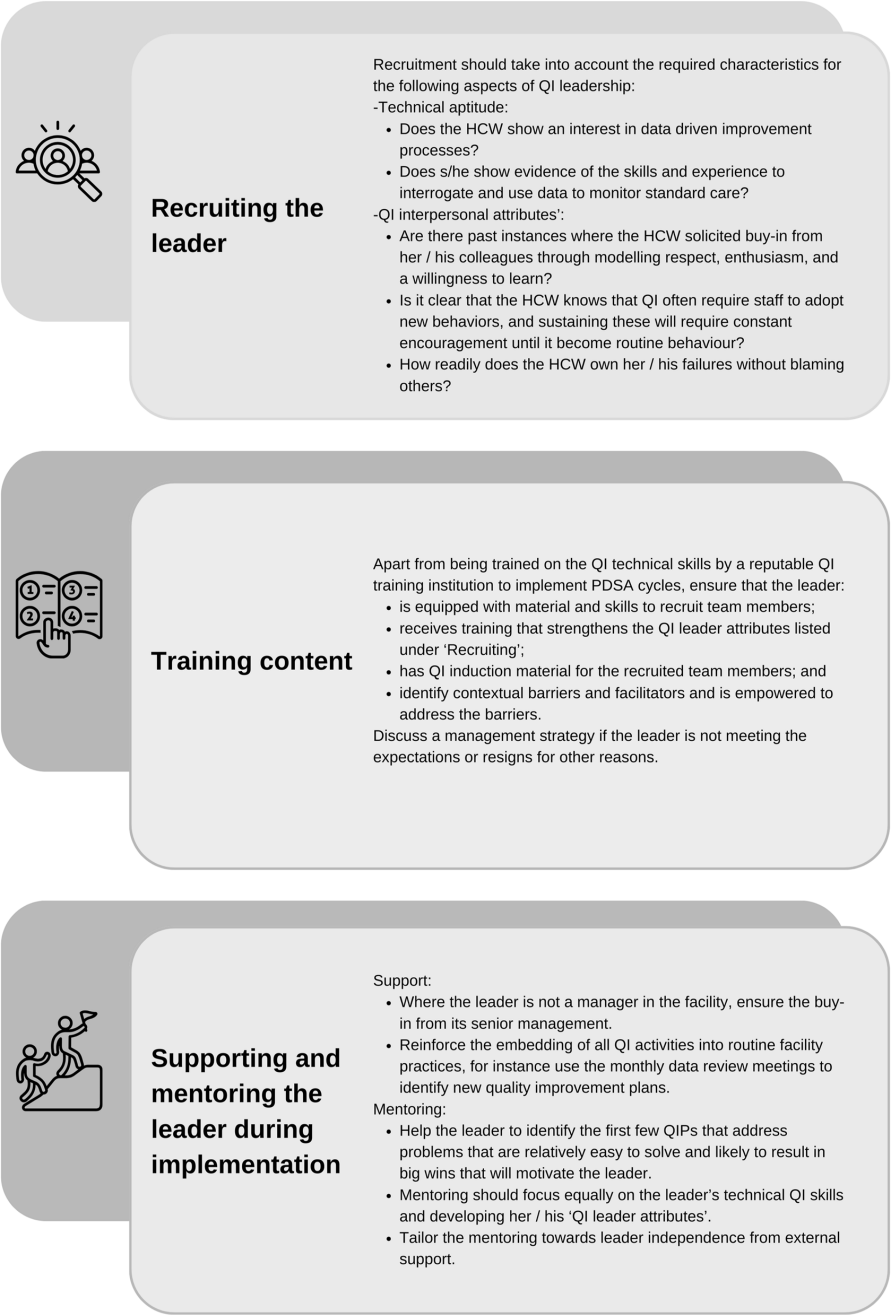
Recommendations to identify and mentor a team leader for a maternal and neonatal QI team

This evaluation ended at about the same time that *Mphatlalatsane* ended, and we do not have data on team sustainability beyond this period. If Butler and colleagues are correct in stating that sustaining a QI team requires a leader who has internalised the approach as standard care, and not someone with only the jargon and technical skills to use it [58], then the six well-performing teams we identified are likely to become sustained teams well beyond the life of the *Mphatlalatsane* programme.

## Strengths and limitations

Conducting the evaluation over 39 months offered longitudinal data that resulted in a nuanced understanding of team leaders’ and QI advisors’ perceptions and experience of leading a QI team. It also allowed a comprehensive view of how leaders shaped team performance. Interviewing the leaders at three time points resulted in a rapport between researcher and participant that was conducive for spontaneous interactions between them. The interviews were conducted in their workplace, and this gave us an appreciation of their realities and better insight into what it took to be a leader. The fieldwork journal offered useful recall of contexts and processes experienced during the fieldwork. This contributed towards a more nuanced interpretation of the data e.g., the fieldwork notes reminded the researchers of the long patient queue at one facility at the time of the interview. This made the participant’s comment that QI takes a backseat because of staff shortage more real and understandable.

Our evaluation is limited by not having conducted baseline interviews with the team leaders immediately after they had set up their teams. Understanding leaders’ expectations and concerns from inception may have provided context for how their leadership played out. It would have been ideal to have all leaders interviewed without members joining the interviews, but the instances where joint interviews happened, were beyond our control as the leaders’ choice led us. We concluded the leader interviews three months before *Mphatlalatsane* ended, but the last advisor interviews (one month before *Mphatlalatsane* ended, did not reveal real changes between implementation and in-field evaluation completion. The study would have benefited from supplementing our qualitative assessment of leader skills and team performance with standardised tools to quantify leaders’ characteristics and skills and teams’ performance. We did not assess the team leaders’ general leader capabilities. Some of the less-well performing QI team leaders may not have had good general leadership skills. Lacking these may have been exacerbated when they were task to become QI leaders.

Gaps in our research to be addressed in evaluating at-scale QI programmes, include quantifying associations between leadership, team size, and team performance; and establishing if facility type relates to team size and performance. Future research should also explore how policymaker endorsement, or not, and it’s cascading downwards across all management levels, impact team leaders and how their teams perform.

### Reflexivity statement

The core research team (WO, XH, TC) were external to the *Mphatlalatsane* programme, with no preconceived ideas of how the QI teams would perform. They had no previous interactions with the QI advisors and team leaders that could have influenced their interpretation of the results. They have sound experience in programme evaluations, and WO and XH are seasoned qualitative researchers.

## Conclusions

Understanding the characteristics and skills of a team leader is crucial to contextualise a QI team’s performance, and as such the outcomes of a QI programme. A leader who embraces QI methods and has the necessary technical skills, is more likely to nurture a team into a well-performing team, than a leader who has no appetite for the methodology. These two attributes must be supported by the leader’s ability to cultivate a learning culture in the team, and tenacity to keep members to task. Careful selection and training of QI leaders will facilitate team performance, and in turn, the effectiveness of the QI programme.

### Supplementary Information

**Additional file 1: **Socio-economic and health indicators of participating districts.

**Additional file 2. **QI advisor and Team leader interview schedules.

**Additional file 3: **Evidence used to rate team performance.

## Data Availability

All the transcriptions and analysed data can be obtained, on reasonable request, from the corresponding author.

## Abbreviations

CHC: Community health centre
HCW: Healthcare worker
MNH: Maternal and neonatal health
NDoH: South African National Department of Health
OPM: Operational manager
PHC: Primary healthcare
QI: Quality improvement
SA: South Africa
