# Supplementary material for: “If you work alone on this project, you can’t reach your target”: unpacking the leader’s role in well-performing teams in a maternal and neonatal quality improvement programme in South Africa, before and during COVID-19
Source: BMC Health Serv Res. 2023 Dec 8;23:1382. doi: 10.1186/s12913-023-10378-x (PMC10709890; doi:10.1186/s12913-023-10378-x)
Supplement: Supplementary file 1 — Additional file 1: Socio-economic and health indicators of participating districts. [file 12913_2023_10378_MOESM1_ESM.docx]

**Additional file 1:** Socio-economic and health indicators of participating districts

|  | **Mpumalanga** | **Limpopo** | **Eastern Cape*** | |
| --- | --- | --- | --- | --- |
| **Population estimates**  **(2018/19)** | 1,743,182 [1] | 138,760 [2] | 1,263,051 [3] | 527,062 [4] |
| **Unemployment rate**  **(2018/19)** | 37% [5] | 39% [2] | 29% [6] | 30% [4] |
| **Health facilities** | 1 tertiary, 2 regional and 8 district hospitals, 15 CHCs, 108 clinics [1] | 1 regional and 6 district hospitals, 8 CHCs, 97 clinics [7] | 2 tertiary, 1 regional and 1 district hospital, 9 CHCs, 39 clinics[8] | 10 district hospitals, 3 CHCs, 59 clinics [8] |
| **Provincial institutional maternal mortality ratio (per 100,000 live births), 2018** | 111 [9] | 106 [9] | 107 [9] | |
| **Live births in facility**  **(2018/19)** | 40,734 [1] | 31,268 [2] | 18,808 [8] | 5,909 [8] |
| **Provincial neonatal mortality rate (per 1,000 live births), 2018** | 10.8 [9] | 12.2 [9] | 12.3 [8] | |

CHC: Community health centre,

* Two districts

**References**

1. Mpumalanga Department of Health. Annual performance plan, 2020-21. Mpumalanga, South Africa; 2020.

2. Mopani District. Annual report, 2018/19. Limpopo, South Africa; 2019.

3. Nelson Mandela Bay District. Profile and analysis, 2020. Eastern Cape, South Africa; 2020.

4. Sarah Baartman District. Profile and analysis, 2020. Eastern Cape, South Africa; 2020.

5. Ehlanzeni District. Profile and analysis, 2020. Mpumalanga, South Africa; 2020.

6. Eastern Cape Department of Health. Annual report, 2018-19. Eastern Cape, South Africa; 2019.

7. Limpopo Department of Health. Annual performance plan, 2020/21-2024/25. Limpopo, South Africa; 2021.

8. Eastern Cape Department of Health. Strategic plan, 2020/21-2024/25. Eastern Cape, South Africa; 2020.

9. South Africa National Department of Health. Strategic plan, 2020/21-2024/25. Gauteng, South Africa; 2020.
