# Supplementary material for: “If you work alone on this project, you can’t reach your target”: unpacking the leader’s role in well-performing teams in a maternal and neonatal quality improvement programme in South Africa, before and during COVID-19
Source: BMC Health Serv Res. 2023 Dec 8;23:1382. doi: 10.1186/s12913-023-10378-x (PMC10709890; doi:10.1186/s12913-023-10378-x)
Supplement: Supplementary file 2 — Additional file 2. QI advisor and Team leader interview schedules. [file 12913_2023_10378_MOESM2_ESM.zip › Supplementary file 2a_Advisor_Interview 1.docx]

**Mphatlalatsane evaluation**

**Advisors: Interview 1 (Feb ‘20)**

Today we want to focus on experiences and perceptions regarding Mphatlalatsane at micro level, that is, what has happened with setting up and supporting the teams until now.

Team functioning

1. What does a typical team look like? Prompts:
2. How many members?
3. Who are they primarily?
4. Is there a division of roles / responsibilities?
5. How exactly were the teams set up? Prompts:
6. Did the facility manager nominated them / asked for volunteers?
7. Was the training they attended the official starting point of their functioning?
8. Thinking back about their training, are there any gaps that should be addressed in refresher training?
9. Please describe their functioning in as much detail as possible: Prompts:
10. How do they manage to find time to conduct PDSA cycles, i.e., is this not disrupting service delivery?
11. How do the team fit within the facility structures and reporting lines?
12. To what extent is their functioning left to themselves and to what extent is it prescribed, either by you or the facility manager? Do they decide when and how often to run a PDSA cycle, and do they decide which issues to address?
13. What are similar and different between the teams? Prompts:
    1. In the same types of facilities within the same catchment area?
    2. In the different types of facilities within the same catchment area?
    3. In the same types of facilities across the different catchment areas?
    4. My understanding is that the E Cape teams have been established pre-Mphatlalatsane; can you please tell us more about this?
    5. Are there successes / challenges that you think relate to the type of facility?
14. What resources, for e.g., stationery, space, time, do they need to do a PDSA cycle? Prompt:
15. Who provides them with it?
16. How important is the facility manager in the success of a QI team?

Team performance

1. How do you rate your teams’ performance at this point? Prompts:
2. Are you happy with how they perform, and can you please tell why you say so - please detail this for each team.
3. Who are the high performing teams and who are struggling at this point, and what is the reason why you think so?
4. What are the enablers and barriers impacting team performance?

Team leader

1. Can you tell me about the leadership within the teams? Prompts
2. Are there formal / informal leaders?
3. Who holds the actual power?
4. What is their vision/s and it is shared by the team?
5. What do they do to mobilise and inspire, and does it impact team functioning and performance?
6. How much of the team performance is shaped by the team leader?

COVID-19

1. How is the pandemic impacting your mentoring and support?
2. How is the pandemic impacting teams’ day-to-day QI activities?
3. How is the pandemic shaping their performance?
