# Supplementary material for: “If you work alone on this project, you can’t reach your target”: unpacking the leader’s role in well-performing teams in a maternal and neonatal quality improvement programme in South Africa, before and during COVID-19
Source: BMC Health Serv Res. 2023 Dec 8;23:1382. doi: 10.1186/s12913-023-10378-x (PMC10709890; doi:10.1186/s12913-023-10378-x)
Supplement: Supplementary file 2 — Additional file 2. QI advisor and Team leader interview schedules. [file 12913_2023_10378_MOESM2_ESM.zip › Supplementary file 2b_Leader_Timepoint 1.docx]

**Mphatlalatsane evaluation**

**Leader Timepoint 1 interview (Apr - May ‘21)**

Participant demographics

1. What is your role in the facility?
2. For how long have you been in (i) nursing, (ii) this facility, (iii) in this role?
3. What is the caseload of maternal and neonate patients in this facility?
4. Have you had any QI experience prior to Mphatlalatsane?

General: Mphatlalatsane and QI methodology

1. What is Mphatlalatsane all about?
2. How was Mphatlalatsane introduced to you?

Prompt: Is your participation voluntary?

1. Did you receive any training? Prompts:
2. How was it decided who should attend?
3. What can you tell me about the training: where, when, content, what was good / bad, did it prepare you sufficiently for the work?
4. Please tell me in as much detail as possible, how it is implemented in your facility. Prompts:
   1. How was the team set up?
   2. How often do you meet, and what happens at these meetings?
   3. Have you any documents that you can show me?
5. At this point, are you positive / neutral / negative about the Mphatlalatsane project, and can you please tell me why so?
6. If it works, why do you think it works?

Team performance

1. How do you rate your team’s performance at this point? Prompts:
2. Are you happy with how the team perform, and can you please tell why you say so?
3. Are they enthusiastic about QI, and why do you say so?
4. Please give an example or 2 of your successes, and share the lessons you have learned so far?
5. What are the enablers and barriers impacting team performance?
6. How important is the leader in the success of your QI team?
7. Sharing successful change ideas with other facilities

COVID-19

1. I want to end off by asking you to reflect on the impact that the pandemic had, and is having on your work MNCH services? Prompts:
2. What was the worst for you?
3. How did you / are you surviving?
4. Impact on services other than MNCH?
5. How has / is it affecting patients’ uptake of MNCH services?
6. Any good at all, that resulted from the pandemic?
